# Pathogenesis of systemic sclerosis associated interstitial lung disease

**DOI:** 10.1177/2397198320903867

**Published:** 2020-03-05

**Authors:** Svetlana I Nihtyanova, Christopher P Denton

**Affiliations:** Centre for Rheumatology and Connective Tissue Diseases, University College London, London, UK

**Keywords:** Fibroblast, autoantibodies, lung fibrosis, pathogenesis, cytokine

## Abstract

Systemic sclerosis is an autoimmune disease leading to vasculopathy and fibrosis of skin and internal organs. Despite likely shared pathogenic mechanisms, the patterns of skin and lung fibrosis differ. Pathogenesis of interstitial lung disease, a major cause of death in systemic sclerosis, reflects the intrinsic disease pathobiology and is associated with distinct clinical phenotypes and laboratory characteristics. The commonest histological pattern of systemic sclerosis–interstitial lung disease is non-specific interstitial pneumonia. Systemic sclerosis–interstitial lung disease pathogenesis involves multiple components, including susceptibility and triggering factors, which could be genetic or environmental. The process is amplified likely through ongoing inflammation and the link between inflammatory activity and fibrosis with IL6 emerging as a key mediator. The disease is driven by epithelial injury, reflected by markers in the serum, such as surfactant proteins and KL-6. In addition, mediators that are produced by epithelial cells and that regulate inflammatory cell trafficking may be important, especially CCL2. Other factors, such as CXCL4 and CCL18, point towards immune-mediated damage or injury response. Monocytes and alternatively activated macrophages appear to be important. Transforming growth factor beta appears central to pathogenesis and regulates epithelial repair and fibroblast activation. Understanding pathogenesis may help to unravel the stages of systemic sclerosis–interstitial lung disease, risks of progression and determinants of outcome. With this article, we set out to review the multiple factors, including genetic, environmental, cellular and molecular, that may be involved in the pathogenesis of systemic sclerosis–interstitial lung disease and the mechanisms leading to sustained fibrosis. We propose a model for the pathogenesis of systemic sclerosis–interstitial lung disease, based on the available literature.

## Introduction

Systemic sclerosis (scleroderma; SSc) is an uncommon disease with prevalence of around 1–2 in 10,000 and some clear associations with sex and age.^1^,^2^ SSc is a prototypic multisystem fibrotic disease that leads to increased extracellular matrix deposition and structural changes in skin and internal organs. The extent of fibrosis varies between patients and across organs within a patient. However, there are common patterns of involvement that allow SSc to be classified into distinct subsets. Most characteristic is the differing extent of skin fibrosis that underpins the classification into limited or diffuse cutaneous subset.^3^

Interstitial lung disease (ILD) is a term that covers a large group of disorders of the lung parenchyma, which involve development of inflammation and/or fibrosis of the lung.^4^ Although many of those disorders are idiopathic, some can develop in the context of connective tissue diseases, including SSc.^5^ In terms of histopathologic and radiographic features, SSc–ILD most often has features of non-specific interstitial pneumonia (NSIP) in up to 78% of subjects, followed by usual interstitial pneumonia (UIP) in up to 36%, while other patterns, such as organising pneumonia are much rarer.^6–9^ In addition, overall survival does not appear to be associated with the histopathologic/radiographic pattern of ILD with no difference in mortality between NSIP and UIP.^6^

ILD is a common complication of SSc. Not all SSc patients undergo lung imaging and high-resolution computed tomography (HRCT) is generally performed only when indicated clinically by presence of dyspnoea, cough and late inspiratory crepitations on auscultation or decline in pulmonary function tests (PFT). As a result, estimation of the prevalence of mild ILD is difficult. On the other hand, prevalence of clinically significant lung fibrosis has been studied extensively, and in incident cohorts, approximately half of the patients are estimated to develop significant ILD, requiring immunosuppressive treatment.^10–12^ Over the last three decades, ILD has become a leading disease-related cause of death in SSc patients.^13–16^ A clear association between extent of ILD and mortality among SSc patients exists and a threshold of 20% extent of ILD on HRCT has been shown to distinguish between patents at high risk that require active treatment and those at low risk who would only require PFT monitoring.^17^ ILD tends to develop early in the disease course^11^ and short disease duration has been identified as one of the strongest predictors of ILD progression.^18^

Although more common in diffuse cutaneous (dc)SSc, ILD can occur in either cutaneous subset.^10^,^11^ It also has been observed in SSc patients carrying any of the SSc-specific hallmark autoantibodies, including anti-RNA polymerase and anti-centromere (ACA) antibodies, although anti-topoisomerase I (ATA; anti-Scl70) positive subjects are at a much higher risk of ILD development compared with any other antibody group.^11^,^19^,^20^ Nevertheless, the risk for ILD development that is related to ATA positivity appears to be independent from cutaneous subset.^11^ This suggests that as well as shared mechanisms, there are differences between skin and internal organs in terms of the development of fibrosis.

SSc is an autoimmune rheumatic disease, and the first abnormalities that are observed as the disease develops are typically the presence of autoantibodies and new-onset or worsening Raynaud’s phenomenon. This offers the opportunity for early diagnosis and has underpinned the concept of pre-scleroderma and the development of proposed criteria for the very early diagnosis of systemic sclerosis (VEDOSS).^21^ That the immune system is central to the development of SSc is also supported by genetic studies that show replication of immune system genetic associations across multiple cohorts. In SSc, it is well-recognised that the first changes that occur in the skin histologically include microvascular endothelial cell activation and later immune cell infiltration. This includes initially cells of the innate immune system (CD68 positive monocyte/macrophages) and later the adaptive immune system with lymphocyte infiltration, including cells with B and T cell surface markers, followed by cells that show an activated immune phenotype, including T follicular helper cells.^22^

Unlike skin, lung fibrosis poses a greater challenge for the researcher. Lung biopsies are very rarely performed in SSc patients, and if done, they are not repeated on multiple occasions and are not possible during the initial stages of disease. For that reason, a substantial part of the information available in the literature on the early stages of SSc–ILD comes from animal models.^23–26^

A plausible model for SSc–ILD pathogenesis is one in which SSc represents a susceptibility phenotype where intrinsic immune mediated or inflammatory injury or extrinsic lung epithelial damage from environmental agents or infection leads to an excessive or exaggerated fibrotic response. The basis for this susceptibility to fibrosis and inability to resolve the fibrotic process once it is established is likely to include additional genetic and local cellular factors. This article explores the basis for these processes. We begin with review of genetic factors that have shown association with SSc–ILD, follow with a discussion of cellular and molecular players, as well as mechanisms leading to sustained fibrosis and finish with a proposed model for the pathogenesis of SSc–ILD, based on the available literature.

## Genetics of SSc–ILD susceptibility

Genetic studies have recently highlighted a number of relevant susceptibility factors for SSc–ILD, although many are shared with other autoimmune diseases, such as rheumatoid arthritis and systemic lupus erythematosus.^27–30^ A recent meta-analysis of genome-wide association studies (GWAS) identifies probably most of the relevant common genetic variants associated with disease susceptibility and starts to link variants with subtypes of disease.^31^ In idiopathic pulmonary fibrosis (IPF), there are additional genetic mechanisms relating to mucin production, including MUC5B polymorphisms,^32^ or possible response to epithelial damage followed by ineffective repair processes, due to changes in telomerase activity and chromosome instability.^33^ Nevertheless, studies have failed to demonstrate association of those with SSc–ILD.^32^,^34–36^

A number of SSc–ILD candidate genes have been identified, although many studies include small numbers or have not been replicated.^37^ The majority are genes for immune response molecules, such as interferon regulatory factor 5 (IRF5),^38–41^ signal transducer and activator of transcription 4 (STAT4),^30^,^42^,^43^ Interleukin-1 receptor-associated kinase-1 (IRAK1),^44^,^45^ CD226^46^ and CD247.^47^ A number of human leukocyte antigen (HLA) region signals have also been identified, including HLA-DRB1, 3 and 5, DQB1 and DPB1,^48–55^ although those associations are strongest with autoantibody specificities. In addition, genetic factors that are non-immune have been indicated, such as connective tissue growth factor (CTGF) polymorphisms.^56–58^ These have not generally been replicated in all cohorts and may not represent a susceptibility gene in all populations, although the discrepancies may be related to differences in the phenotype definitions between patients from different centres. Modern genetic sequencing approaches, including direct sequencing, may eventually shed light on this by identifying rare functional variants that directly affect pathogenesis of SSc–ILD.^59^

## Cellular pathogenesis

The cellular pathogenesis of SSc–ILD reflects the complex nature of the disease that involves cells of the innate and adaptive immune system, vasculature and connective tissue as well as specialised lung structures, including alveolar epithelial cells (AECs). Inflammation and immune activation are early events and include infiltration of monocytes and macrophages within the lung parenchyma and accumulation of increased inflammatory cells in the alveolar space.^60^ These phagocytic species reflect the need to remove toxic and infective material from the airspaces and highlight the lung as an environment where effective tissue homeostasis and resolution of injury are central to normal function. T and B cell infiltration follows and studies have demonstrated increased numbers of activated CD8+T lymphocytes, which produce profibrotic factors, including IL4, Oncostatin M and may activate latent transforming growth factor beta (TGF-β).^61^ These cells can be sampled by bronchoalveolar lavage (BAL), which has provided valuable insight into the pathogenesis of ILD.^62–65^ Granulocytosis has been found in two thirds of the patients with SSc–ILD.^63^ Although neutrophilia and eosinophilia can also be observed and earlier studies suggest they predict future progression,^6^,^62^,^64^ more recent publications show that the cellular profile of BAL fluid does not associate with SSc–ILD prognosis and is therefore not routinely performed in SSc patients.

AEC injury is another important element of the SSc–ILD pathogenesis and AECs may be damaged by environmental stimuli or from local inflammation.^60^,^66^ Instead of repair from proliferation of type II cells, damage to type I cells is followed by migration of fibroblasts that lead to fibrotic tissue development.^67^ There are also important populations of specialised epithelial cells that may produce surfactant proteins that are essential for normal physiological lung function and repair of lung injury.^68^ The vascular compartment is critical for gas exchange and includes specialised cells within the blood vessel wall. Endothelial cells provide a critical barrier function to facilitate gas exchange as well as the large surface area essential for effective oxygen transfer. Smooth muscle cells, adventitial fibroblasts and specialised pericytes are also involved in response to tissue injury and may contribute to fibrosis.^69^

Fibroblasts and myofibroblasts are the driving cells for scar formation and these can arise from multiple lineages including trans-differentiation from endothelial cells, epithelial cells or pericytes.^70^,^71^ They may be recruited from a number or circulating precursors including fibrocytes and monocytes.^72^ Expansion of local interstitial fibroblasts and resident lung progenitor mesenchymal cells are also important.^73^,^74^

## Key cytokines and molecular pathways that drive SSc–ILD

Cytokines and growth factors provide the intercellular mediators that co-ordinate and regulate tissue repair and the activation of the cellular players that are described above. It is likely that cytokines act in context and that multiple cell types are regulated by paracrine, autocrine and intracrine processes.^75^ TGF-β is the major regulator of connective tissue growth and repair in embryonic development and postnatally. It also is well placed to coordinate post-natal response to tissue injury. It is preformed and sequestered in the extracellular matrix and activated when needed through a number of mechanisms.^76^ Some of these such as integrin dependent activation may be especially relevant to lung injury and fibrosis.^77^

Other cytokines are produced by lung inflammatory and epithelial cells. These include ubiquitous growth factors such as basic fibroblast growth factor (bFGF) and vascular endothelial growth factor (VEGF) and chemokines such as CCL2, CCL7 and CCL18.^78–83^ There is considerable interest in these mediators as potential targets for treating fibrosis and this is important because they can be produced by and action multiple relevant cell types. These are not generally disease specific and represent mediators of tissue repair and responses to cellular injury. Chemokines are emerging as major candidate mediators in SSc–ILD based upon the ability of damaged lung tissue, especially epithelial cells, to produce chemokines such as CCL2 that may then modulate inflammation and leucocyte migration.^78^,^79^ Other chemokines may derive from platelets, such as CXCL4 (ILD4)^84^,^85^ or other infiltrating cells, including immune cells (CCL18). Levels of CCL18 and CCL2 have been associated with outcome in observational cohorts and clinical trials.^78^,^79^,^81^,^82^

IL6 has been shown to be important in ILD locally and systemically. Recent studies point towards a particularly important role in early stages of SSc–ILD and it may link inflammation and fibrosis.^86^ Intracellular signalling pathway for IL6 and TGF-β converge, including STAT3, and also cellular interaction may link fibroblast derived IL6 and other cell types such as macrophages.^87^ There is a growing appreciation of the potential important role of macrophages in fibrosis and that the diverse functional properties and ontogeny of macrophages in the lung may be important in pathogenesis and therapy of ILD.^88^ The recent licencing of nintedanib shows that blocking intracellular signalling for multiple ubiquitous growth factors can be very effective.^89^ Similarly, the data from the tocilizumab trials suggest that Interleukin 6 receptor (IL6R) blockade can also be effective in those SSc patients most at risk of early progression of ILD.^90–92^

Lung injury is associated with activation of the coagulation pathway and this can result in release of products of the coagulation pathway that are profibrotic. This includes thrombin and later stage factors, such as factor XIII. The latter is a transglutaminase that may link the late stages of coagulation after injury and may promote TGF-β activation via secondary effects on thrombospondin, a major activator of TGF-β in animal models.^93^

## Lessons from preclinical models

The complex nature of SSc and its hallmark clinical heterogeneity have been a challenge for the development and validation of preclinical models. Not all animal models of SSc develop pulmonary fibrosis. The most widely used models for SSc–ILD are the bleomycin lung injury model and a number of genetic models, including Fra-2, Fli1, uPAR and TbRIIΔk-fib. The bleomycin model is well established in ILD research.^94^ Although different protocols exist, which include varying doses and routes of administration (transoral, endotracheal, subcutaneous, intravenous and intraperitoneal), the fibrotic process follows approximately the same course. Acute inflammation develops within the first 7 days, followed by fibrosis 1–2 weeks after bleomycin administration.^94^ In wild type mice, the fibrosis generally resolves. Use of genetically modified mice that develop mild spontaneous ILD can show more extreme changes in response to lung injury and have helped to define critical mediators or candidate pathways for attenuation.^95^,^96^ A transgenic mouse model with altered TGF-β signalling in fibroblasts (TbRIIΔk-fib) has been shown to develop ILD in approximately 25% of the animals from 6 weeks^97^,^98^ and in response to minor epithelial lung injury and may represent a model for milder SSc-associated ILD.^95^,^96^ This mouse strain also develops other relevant abnormalities in the skin and vasculature.^23^ The Fra-2 transgenic mouse offers another model for SSc and from 12 weeks the animals develop skin and lung fibrosis.^99^,^100^

## Mechanisms that sustain fibrosis

In SSc, it seems likely that changes in the skin reflect activity of the proinflammatory process and this is reflected by the progression that occurs in the first 12–24 months. This is followed by stabilisation or regression when the normal biology of wound healing prevails and leads to softening of the skin and improvement in the mRSS. It seems likely that to some extent, the progressive phase occurs throughout affected organs, although the timing and extent may differ. In particular, there may be inherent differences between organs in the extent to which regression and de-remodelling of scarred tissue can occur with later regeneration of specialised structures and organs.

**Figure 1.**
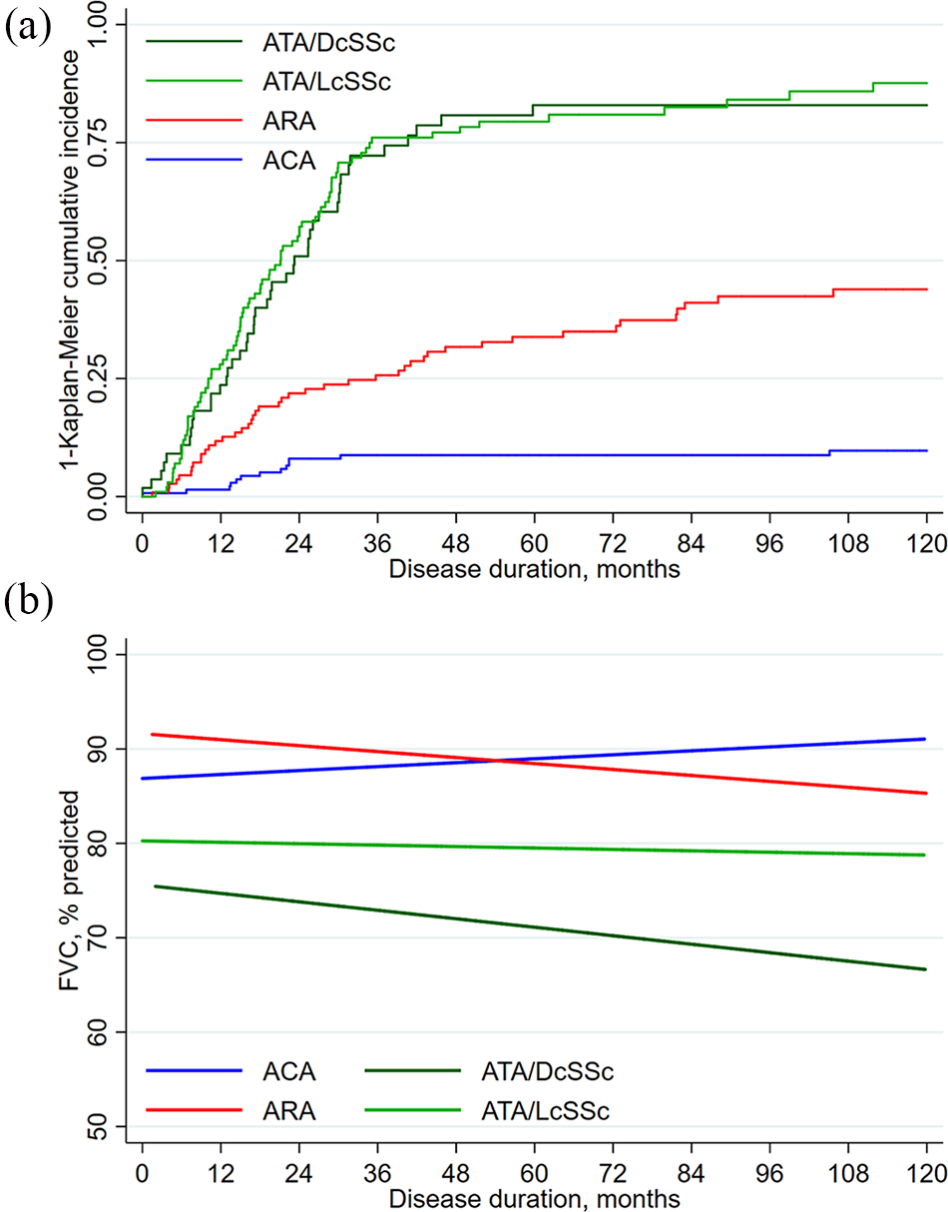
Development and progression of SSc–ILD suggests contrasting drivers in disease subsets: (a) Time to clinically significant pulmonary fibrosis and (b) average FVC change over time in subgroups by antibodies and subset. Autoantibody and skin subset appear to determine the development and progression of SSc–ILD. Thus, some antibodies are associated with early development of extensive disease,^17^ with ATA having the highest risk. (a) Time to development of clinically significant ILD in 403 SSc patients, positive for the three most common scleroderma-specific autoantibodies, anti-centromere (ACA), anti-topoisomerase I (ATA) and anti-RNA polymerase antibody (ARA). While in their great majority, ACA+ patients develop limited cutaneous and ARA+ diffuse cutaneous subset of SSc (97% and 92% in this cohort), ATA+ subjects can present with either subset (35% limited and 65% diffuse in this cohort), which does affect their overall survival, but not their risk of significant ILD. (b) Modelling FVC changes over time in 297 SSc–ILD patients, who were positive for either ACA, ATA or ARA. Disease progression, based on modelled FVC trajectory, show relevant ANA associated differences. Thus, ARA and ATA overall have similar later-stage rate of progression, but at different levels of impairment, reflecting higher risk of early severe ILD in ATA. This later stage progression likely depends on factors outlined in Figure 1 and may also reflect systemic fibrotic activity, as the slope of decline for ATA+ cases is greater in those with diffuse compared with limited skin involvement.

In the lung, it is likely that once fibrosis is established, with disorganised lung architecture and structural changes, this may be essentially irreversible. This would fit with observed changes in skin and lung over time that have recently been described in SSc patients.^11^ High-risk cases develop severe or extensive ILD relatively early and the hazard of clinically significant ILD development is highest in the first 24–36 months of the disease, rapidly declining thereafter. This is also the time period when mRSS would peak for the majority of SSc patients. While skin then improves for over 80% of the patients,^101^ ILD may stabilise or progress, and the rate of progression is determined by the extent of fibrosis, which was used as the basis of the Goh staging system.^17^ The trajectories for forced vital capacity (FVC) change over time for a large cohort of SSc cases under long-term regular review are shown in Figure 1. The high-risk cases with ATA experience a significant drop in FVC much earlier than patients with other SSc-specific antibodies. ATA is also associated with the slowest rate of skin improvement.^11^ The rates of change in FVC in the later stages of disease appear similar between groups and likely reflects shared pathogenic mechanisms for worsening. These include recurrent lung injury that could be essentially the level of normal environmental challenge that healthy individuals experience, but that in SSc there is greater tendency to fibrosis and less ability to recover without fixed scar. Other factors could include microaspiration with damage from bile salts and acid, despite maximal anti-reflux treatments.^102^,^103^ Mechano-sensing due to stiffened lung tissue is also important and this has been shown to drive myofibroblast differentiation via a number of pathways including myocardin-related transcription factor-A (MRTF-A) pathway and yes-associated protein (YAP).^104^ All of these mechanisms may represent molecular targets for treatment in the future.

## Phases of pathogenesis for SSc–ILD

**Figure 2.**
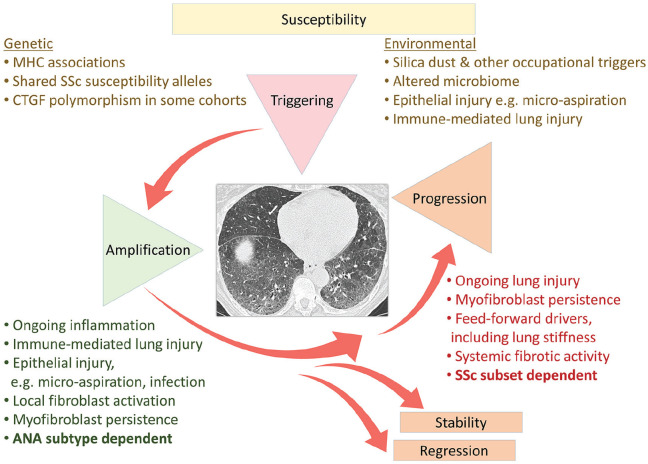
Phases of development and progression of lung fibrosis in systemic sclerosis (SSc–ILD). Schematic illustrating the three independent phases in pathogenesis of SSc–ILD that reflect different susceptibility within the SSc subgroups based upon skin extent and ANA reactivity. In a susceptible patient, triggering events that may reflect lung injury or intrinsic disease-related immune mechanisms lead to interstitial inflammation and fibrosis. This may then extend and become clinically meaningful through similar mechanisms. Outcome of progression, stability or regression will be affected by systemic disease features, including SSc subset, ANA and intrinsic lung fibrotic mechanisms such as tissue stiffness and structural changes to lung architecture.

The development and progression of ILD in SSc can be envisioned into the main stages that are outlined and summarised in Figure 2.

### Early phase – susceptibility and triggering

Like in most complex diseases, aetiopathogenesis of SSc–ILD is likely to be complex and multifactorial and to involve triggering events occurring by chance in a susceptible individual. This susceptibility is likely to be at least genetic or epigenetic, but environmental cofactors may also be relevant, such as the lung microbiome.^105^,^106^ Environmental influences are likely to include those that may initiate or trigger the disease and others that modulate progression. The latter may be especially relevant to ILD where there might be susceptibility to tissue damage and a predilection to fibrotic scarring in response to recurrent or persistent lung injury due to environmental chemical exposure, recurrent microaspiration^102^,^107^ or episodes of infection.^108^,^109^

It seems likely that the early stages of lung involvement in SSc, especially in those patients with the highest risk of ILD, will reflect the same processes observed in other SSc-related organ complications, including endothelial activation and T and B cell infiltration. Later neutrophils predominate in BAL fluid and may reflect the extent of lung damage.^63^ High-resolution CT patterns support the importance of early inflammation with amorphous ground glass change although this may represent fine fibrosis rather than pure inflammation even in early stage disease.^9^

Cohort studies have highlighted that early SSc is often associated with loss of lung function, especially in the diffuse cutaneous subset, and this is supported by data from several recent clinical trials recruiting very early stage cases. Thus, in RISE-SSc even with average disease duration of less than 9 month, there was evidence of clinically meaningful decline in FVC% predicted over 52 weeks.^110^ Likewise, two placebo-controlled trials of tocilizumab that enrolled early stage (less than 2 years average disease duration) dcSSc subjects with evidence of increased acute phase markers, showed very marked overall decline in FVC in the placebo arm, although interestingly this was largely prevented by therapeutic IL-6 receptor blockade in the tocilizumab treatment arms.^90–92^ This highlights the progressivity of mild early ILD in dcSSc and the potentially important role of IL6 as a driver of progression. These interventional trial data are supported by observational data from two cohorts that also identify serum IL6 as a significant predictor of lung function decline and death in early stage or milder ILD.^86^,^111^

The importance of early inflammation is supported by preclinical models such as the bleomycin model of ILD that has been used in many laboratory studies. Links between anti-nuclear antibody (ANA) pattern and risk of development of severe ILD also point to the importance of immune mechanisms, but also imply that these may differ between the ANA-defined subsets in SSc. For some high-risk antibodies, this seems to be independent of the extent of skin fibrosis, as it is not impacted by SSc subset once the onset of disease is classified by first non-Raynaud’s manifestation.^11^

The consequence of early inflammation is the development of an activated fibroblast population that has a marked pro-fibrotic phenotype. This includes a gene and protein expression profile of TGF-β activation. It is likely that distinct subpopulations of fibroblasts in the lung contribute to the development of fibrosis and these are likely to have distinct origins.^67^ They include cells derived from local fibroblast activation and proliferation as well as cells derived from other lineages, notably epithelial cells and endothelial cells as well as progenitor populations including pericytes. Preclinical experiments have defined an essential role for resident lung fibroblasts in regulating the fibrotic process and confirmed that this is dependent upon intact TGF-β signalling.^96^

### Established phase – progression and failed resolution

Clinically, there is evolution from the early inflammatory lesion towards a more fibrotic phenotype. This equates with the development of a typical NSIP pattern of ILD that may be cellular or fibrotic.^6^ The drivers of this process are likely to include ongoing inflammation and the interplay between the innate and adaptive immune system and fibroblasts that leads to increased matrix deposition. It seems likely that this phase of pathogenesis is influenced by the activity of the disease process and determines whether patients remain with mild and more stable ILD or evolve into a more extensive disease. Factors that determine this may include intensity of the inflammatory process,^63^,^64^ recruitment of profibrotic cell populations, including circulating fibrocytes,^64^,^72^ and failure of apoptosis of myofibroblasts that has been demonstrated to be a key mechanism in experimental mouse models of SSc–ILD.^95^ Many of these changes may reflect a background profibrotic susceptibility phenotype in SSc that links to the pattern of diseases, including ANA-defined subgroups.^11^,^112^ Factors such as altered microbiome or recurrent aspiration and lung injury may become relevant at this stage and determine whether lung disease progresses.^105–108^ Local activation of fibrotic pathways and recruitment of feed-forward loops involving TGF-β dependent pathways such as mechano-sensors including MRTF-A and YAP–TAZ may be involved.^104^,^113^

### Late phase – severe fibrosis in a subset of patients

Clinically, the most important stage of ILD in SSc is the established progressive phase. It has been noted that not all cases progress and in fact some remain remarkably stable. However, those that develop more extensive disease have a poor outcome due to ILD and other complications, such as pulmonary hypertension.^114^ In this phase, the risk of progression has been demonstrated to reflect the extent of disease and damage.^17^ This may be the result of mechanical stiffness and altered lung structure, implying that a pattern of progression more akin to IPF may be relevant, albeit less rapidly progressive overall.^115^ This is the phase of disease recruited into most SSc–ILD trials and is probably the most appropriate stage to tackle underlying fibrotic pathways that are shared across different forms of ILD. This would be in keeping with data from emerging clinical trials, such as INBUILD, that show treatment effect is much greater in more severe and progressive cases of ILD than in a mixed cohort of SSc–ILD, although there is almost complete agreement between the proportional impact of anti-fibrotic treatment with nintedanib.^89^,^116^ This more extensive and progressive disease is more likely to show major architectural disruption and more UIP-like pattern on CT or lung biopsy. Recurrent infection and aspiration are likely to be major drivers and some cases may develop pleuroparenchymal fibroelastosis that is especially associated with recurrent infection.^117^

## Concluding remarks

SSc–ILD remains an important challenge, but is now starting to reveal key mechanisms and potential therapeutic avenues. The recent success of targeting tyrosine kinase activity linked to specific receptors for several major growth factors – platelet-derived growth factor (PDGF), VEGF, fibroblast growth factor (FGF) and colony stimulating factor 1 (CSF1), as well as Src family kinases for treatment of ILD.^118^ Initial trials were in IPF, but more recently, nintedanib was shown to have a major effect on progressive lung fibrosis of other causes. In SSc, treatment with nintedanib resulted in a comparable reduction in rate of decline of lung function in SSc–ILD patients.^89^ The latter is a much less progressive condition overall and so demonstration of treatment effect is a major confirmation of the antifibrotic efficacy of nintedanib. This also suggests that the relevant fibrotic processes, shared with more progressive forms of ILD, are less central in SSc. Conversely, recent results from two studies of anti-IL6R show more marked treatment effect on lung function in a subgroup of early stage active dcSSc at particular risk of ILD.^90–92^ This likely targets the earlier stage pathogenic pathways and mechanisms that underlie the prevalent early progressive phase of ILD in SSc whereas a later less progressive but more IPF like fibrotic mechanisms is targeted by nintedanib.^118^,^119^ Thus, reverse translation is likely to shed important light on stages and mechanisms of pathogenesis for SSc–ILD in the future.
